# Do Predictors of Career Success Differ between Swedish Women and Men? Data from the Swedish Longitudinal Occupational Survey of Health (SLOSH)

**DOI:** 10.1371/journal.pone.0140516

**Published:** 2015-10-26

**Authors:** Anna Nyberg, Linda L. Magnusson Hanson, Constanze Leineweber, Gunn Johansson

**Affiliations:** 1 Stress Research Institute, Stockholm University, Stockholm, Sweden; 2 Department of Psychology, Stockholm University, Stockholm, Sweden; Radboud University Nijmegen, NETHERLANDS

## Abstract

The aim of this prospective study was to explore predictors of objective career success among Swedish women and men, focussing on gender differences. Data were drawn from the 2008 and 2010 waves of the Swedish Longitudinal Occupational Survey of Health (SLOSH) with a total of 3670 female and 2773 male participants. Odds ratios and 95% confidence intervals for job promotion and an above-average salary increase between 2008 and 2010 were obtained through binary logistic regression analyses. Individual and organisational factors measured in 2008 were used as predictors in analyses stratified by sex. Mutual adjustment was performed for these variables, as well as for labour market sector and staff category at baseline. In both sexes, younger age predicted both job promotion and an above-average salary increase. Job promotion was also in both sexes predicted by being part of decision-making processes, having conflicts with superiors, and being eager to advance. Furthermore, promotion was predicted by, among men, being educated to post-graduate level and having an open coping strategy and, among women, working >60 hours/week. An above-average salary increase was predicted in both sexes by having a university education. Postgraduate education, having children living at home, and being very motivated to advance predicted an above-average salary increase among women, as did working 51–60 hours/week and being part of decision-making processes in men. Gender differences were seen in several predictors. In conclusion, the results support previous findings of gender differences in predictors of career success. A high level of education, motivation to advance, and procedural justice appear to be more important predictors of career success among women, while open coping was a more important predictor among men.

## Introduction

It is well known that a higher status in society is associated with better health, and the uneven distribution of financial resources between sexes has been shown to (partly) explain poorer wellbeing and health among women compared to men [1–5]. Like women in many other countries Swedish women have poorer wellbeing and health than men, and there is a substantial income gap between sexes. In 2011 Swedish women earned 14% less than men [6–10]. Although labour market participation among Swedish women is high, women work in professions with lower wages and fewer opportunities for career development and are under-represented in senior management to a higher extent than women in several other countries [6,11,12]. Better knowledge regarding the predictors of career success, possibly leading to higher status and better health among (Swedish) women is therefore essential. In a review of the international literature on predictors of objective career success (promotion and salary) Ng, Eby, Sorensen et al. found that the association between human capital variables (i.e. education and hours worked), and salary were stronger for women than men, whereas they found no gender differences in predictors of promotion [13]. However, results from two recent prospective studies indicate that human capital variables are better rewarded in terms of salary for men than women [14,15]. To our knowledge, no prospective study of gender differences in predictors of objective career success has been conducted using a contemporary representative sample of the Swedish working population.

The objectives of the present prospective study were to assess 1) whether organisational and individual factors predicted a) job promotion and b) an above-average salary increase between 2008 and 2010 in Swedish women and men, and 2) whether the associations differed by sex.

## Materials and Methods

### Study sample

A prospective study was performed using questionnaire data from the Swedish Longitudinal Occupational Survey of Health (SLOSH). SLOSH is a nationally representative cohort of the Swedish working population, which started in 2006 as a follow-up to the 2003 Swedish Work Environment Surveys (SWES) conducted by Statistics Sweden. Subsequent follow-ups in SLOSH are conducted every second year and the participants are followed by means of a postal self-completion questionnaire in two versions: one for those currently in paid work at least 30% of full time and one for those in paid work fewer hours or who are not working at all. A more detailed description of the study can be found elsewhere [16]. The present study is based on the respondents from SLOSH 2008 and 2010 (follow-ups of SWES 2003 and 2005). The response rate in 2008 was 61.1% (n = 11441). Out of those, 76.7% also participated in 2010 (n = 8771). Only respondents who identified themselves as in paid work in both waves were included in our analyses (n = 6,580). Respondents to the 2008 and 2010 questionnaire differed from non-respondents to SLOSH on a number of characteristics. In comparison to non-respondents, a somewhat higher proportion of respondents were women, married, born in Sweden, had university education, worked in the governmental sector and their mean age was higher. The participants received written information about the study and, in accordance with Swedish regulation and practice, responding to and returning the survey indicated informed consent. The Regional Research Ethics Board in Stockholm approved the study.

### Variables

Respondents who reported having a higher position in 2010 than 2008 were categorised as having been promoted, and those who reported an unchanged position as not promoted. Respondents with a lowered position in 2010 relative to their position in 2008 (n = 202) were excluded from the analyses of promotion. Yearly salary in SEK was derived from national registers and includes all taxable income (e.g. salary, parental benefits, sickness benefits, pensions, and unemployment benefits). Respondents with a decreased salary (n = 1065) were excluded from analyses of salary increase since these individuals may have started working fewer hours due to a variety of reasons, such as parenthood, retirement, or sickness. The mean increase in salary among men and women included in our study between 2008 and 2010 was 50000 SEK. Any value higher than this threshold was categorised as an above-average salary increase. A higher cut-off was not used in our analyses because it would have included too few women. Labour market sector was categorised into private, public, and other, and adjusted for in the regression analyses in addition to staff category, categorised into blue-collar, white-collar, manager, and other. Age was divided into the categories 19–30, 31–40, 41–50, and 51–70 years. Whether children were living at home or not was included in the analyses as a dichotomous variable. Educational level was analysed in three categories: up to 12 years of school, university education, and postgraduate education. Number of hours worked was categorised into ≤40, 41–50, 51–60, and >60 hours per week. Procedural justice consists of 7 items reflecting whether the decision-making procedures are accurate, correctable, consistently applied, and whether the procedures include opinions from the people involved. The 5 response alternatives range from completely agree (1) to completely disagree (5). The internal consistency of the sum index was high (Cronbach’s alpha, α = 0.91). The sum score was divided into quartiles with the best quartile categorised as high procedural justice and the others comprising the reference category (the cut off for poor procedural justice was 14 points) [17]. Taking part in decision-making processes was measured with the question “To what extent are you involved in decision-making at your workplace?” The response alternatives “to a very great extent” and “to a great extent” were categorised as to be part of decision-making and the response alternatives “to a small extent” and “to a very small extent/not at all” formed the reference categories. Having a manager who listens to you was measured with the question: “Does your manager genuinely listen to what you have to say?” The response alternatives “to a very great extent” and “to a great extent” were categorised as a manager who listens and the response alternatives “to a small extent” and “to a very small extent/not at all” formed the reference categories. Conflicts with superiors were measured with the question: “In the past two years, have you been involved in any kind of conflict with bosses at your workplace?” with the response alternatives yes or no. The question regarding coping strategy is worded as: “During the past two years, when you have felt steamrollered or unfairly treated by your manager/managers, how have you reacted?” “Immediately made clear and clearly showed my feelings” and “Suggested a compromise or other solution” formed the open coping strategy and “Kept quiet and brooded over it” and “Taken it out on my family/those closest to me” the covert coping strategy. Using any coping strategy “always” or “most of the time” indicated a use of this coping strategy [18,19]. Motivation to advance professionally was categorised into “not at all”, “to some extent”, and “very eager” to advance.

### Statistical methods

In order to assess whether organisational and individual factors predicted job promotion and an above-average salary increase, our first objective, we analysed data with two separate logistic regression models, each stratified by sex. We obtained odds ratios and 95% confidence intervals of each outcome (job promotion and an above average salary increase respectively) in 2010 for possible predictors measured in 2008. The predictors were socio-demographic variables (age and having children living at home), human capital variables (educational level and hours per week in paid work), organisational variables (procedural justice and being part of decision-making processes), relationship with manager (having a manager who listens to you and conflict with manager), and coping strategies and motivation (open coping, covert coping, and being eager to advance). The predictors were all included simultaneously in each of the two models, and were thus mutually adjusted for each other. We furthermore adjusted for labour market sector and staff category at baseline. To investigate whether the associations differed by sex, our second objective, we tested for interactions on the additive and multiplicative scales between sex and the potential predictors. Additive interaction was measured with relative excess risk due to interaction (RERI) in STATA V.13 [20]. Each multiplicative interaction term was added to the full model in separate non-stratified logistic regression analyses. Statistical differences in the distribution of variables between men and women were estimated with chi-square tests. All analyses except the tests of additive interactions were conducted with SPSS version 21.

## Results

Men and women were promoted to the same extent over the study period, but men more often received above-average salary increases. Equally, men more often worked within the private sector and as managers. The proportion of women who were very eager to advance was almost equal to that of men, but there was also a higher percentage of women than men who were not at all eager to advance. In total, 12 percent of the women and 24 percent of the men reported working at least 51 hours a week. Reporting high procedural justice, being part of decision-making processes at work, having conflicts with superiors, and using open coping strategies were more common among men, and covert coping strategies were more common among women (see Table 1).

**Table 1:** Descriptive statistics in numbers (percentages) according to included study variables among participants (in paid work who answered the SLOSH questionnaires in 2008 and 2010).

	Women	Men	
	n (%)	n (%)	*p*
Number of participants included (2008 and 2010)	3670	2773	
Yearly salary (SEK)			
2008	275230	359780	< .001
2010	306370	400940	< .001
*Outcomes*			
Promoted over the past two years (measured in 2010)	445 (13.4)	371 (12.1)	n.s.
Salary increase above average between 2008 and 2010	936 (25.5)	1069 (37.1)	< .001
*Covariates*			
Sector (measured in 2008)			< .001
Private	1093 (32.9)	1851 (70.9)	
Public	2147 (64.6)	703 (26.9)	
Other	84 (2.5)	58 (2.2)	
Staff category (2008)			< .001
Blue–collar	1344 (39.7)	1073 (40.8)	
White–collar	1603 (47.4)	1062 (40.4)	
Manager	273 (8.1)	405 (15.4)	
Other	163 (4.8)	87 (3.3)	
*Socio-demographic variables*			
Age (2008)			n.s.
19–30	186 (5.1)	119 (4.3)	
31–40	686 (18.7)	535 (19.3)	
41–50	1145 (31.2)	814 (29.4)	
51–70	1653 (45.0)	1305 (47.1)	
Have children living at home (2008)	1838 (50.7)	1395 (50.8)	n.s.
*Human capital variables*			
Educational level (2008)			< .001
12 years of school	1787 (48.7)	1658 (59.8)	
University education	1837 (50.1)	1064 (38.4)	
Postgraduate education	44 (1.2)	50 (1.8)	
Hours/week in paid work (2008)			< .001
≤ 40 hours	1260 (39.7)	443 (17.9)	
41–50 hours	1526 (48.1)	1440 (58.2)	
51–60 hours	312 (9.8)	432 (17.5)	
> 60 hours	73 (2.3)	159 (6.4)	
*Organisational variables*			
High procedural justice (2008)	675 (20.6)	631 (24.2)	< .001
Part of decision-making processes at workplace (2008)	1872 (53.7)	1622 (60.6)	< .001
*Relationship with manager*			
Manager who listens (2008)	2386 (73.1)	1722 (72.4)	n.s.
Conflict with manager (2008)	716 (20.2)	646 (24.0)	< .001
*Coping and motivation*			
Open coping (2008)	1987 (61.0)	1671 (65.7)	< .001
Covert coping (2008)	402 (12.3)	106 (4.2)	< .001
Eager to advance (2008)			< .05
Not at all	1241 (40.6)	817 (36.6)	
To some extent	1601 (52.4)	1247 (55.8)	
Very	216 (7.1)	171 (7.7)	

*p* level of significance in chi-square tests of differences between women and men

*n*.*s*. non-significant

### Socio-demographic variables

As shown in Tables 2 and 3 lower age was associated with higher odds of both promotion and receiving an above-average salary increase. Having children living at home was not a significant predictor of promotion in either sex, but was positively associated with an above-average salary increase among women. Further analyses showed that adjustment for taking days off work to care for sick children in the past 12 months (measured in 2008) made the relationship between having children living at home and an above-average salary increase among women non-significant (data not shown).

**Table 2:** Odds ratios (OR) and 95% confidence interval (95% CI) of promotion measured in 2010 for predictors measured in 2008. Data are derived from the SLOSH study containing 3670 female and 2773 male participants.

	Women	Men
	OR (95% CI)	OR (95% CI)
*Socio-demographic variables*		
Age (ref: 51–70 years)		
19–30 years	**3.70 (2.05; 6.65)**	**3.06 (1.60; 5.87)**
31–40 years	**2.36 (1.56; 3.56)**	**2.68 (1.78; 4.05)**
41–50 years	**2.04 (1.40; 2.99)**	**2.02 (1.36; 2.99)**
Children living at home (ref. no)		
Yes	1.25 (0.91; 1.71)	1.04 (0.76; 1.42)
*Human capital variables*		
Educational level (ref: 12 yrs of school)		
University education	1.08 (0.80; 1.47)	1.29 (0.93; 1.80)
Postgraduate education	2.19 (0.94; 5.10)	**2.44 (1.02; 5.86)**
Hours/week in paid work (ref: ≤ 40 hours)		
41–50 hours	1.29 (0.94; 1.78)	1.01 (0.64; 1.58)
51–60 hours	1.61 (1.00; 2.60)	1.34 (0.78; 2.29)
> 60 hours	**2.62 (1.14; 6.04)**	1.70 (0.82; 3.55)
*Organisational variables*		
Procedural justice (ref: no)		
Yes	1.37 (0.97; 1.94)	0.86 (0.58; 1.28)
Part of decision-making processes (ref: no)		
Yes	**1.40 (1.04; 1.90)**	**1.82 (1.31; 2.54)**
*Relationship with manager*		
Manager who listens (ref: no)		
Yes	1.37 (0.96; 1.96)	1.32 (0.90; 1.93)
Conflict with manager (ref: no)		
Yes	**1.41 (1.03; 1.94)**	**1.69 (1.22; 2.33)**
*Coping strategies and motivation*		
Open coping (ref: no)		
Yes	0.92 (0.68; 1.26)	**1.48 (1.06; 2.07)**
Covert coping (ref: no)		
Yes	1.41 (0.92; 2.18)	1.39 (0.68; 2.83)
Eager to advance (ref: not at all)		
To some extent	**1.78 (1.28; 2.48)**	**2.17 (1.48; 3.18)**
Very	**5.04 (3.19; 7.94)**	**3.79 (2.23; 6.42)**

Values in bold type indicate significant results (p<0.05).

Adjusted for labour market sector and staff category at baseline.

**Table 3:** Odds ratios (OR) and 95% confidence interval (95% CI) of above-average salary increase between 2008 and 2010 for predictors measured in 2008. Data are derived from the SLOSH study containing 3670 female and 2773 male participants.

	Women	Men
	OR (95% CI)	OR (95% CI)
*Socio-demographic variables*		
Age (ref: 51–70 years)		
19–30 years	**6.47 (4.11; 10.17)**	**5.06 (2.98; 8.59)**
31–40 years	**2.93 (2.13; 4.02)**	**1.84 (1.35; 2.49)**
41–50 years	**1.79 (1.34; 2.40)**	**1.64 (1.24; 2.17)**
Children living at home (ref: no)		
Yes	**1.40 (1.09; 1.80)**	1.06 (0.84; 1.34)
*Human capital variables*		
Educational level (ref: 12 years of school)		
University education	**1.54 (1.21; 1.96)**	**1.59 (1.24; 2.04)**
Postgraduate education	**3.72 (1.73; 8.01)**	1.31 (0.60; 2.90)
Hours/week in paid work (ref: ≤ 40 hours)		
41–50 hours	1.13 (0.88; 1.44)	0.86 (0.64; 1.17)
51–60 hours	1.36 (0.92; 2.01)	**1.52 (1.04; 2.21)**
> 60 hours	1.28 (0.60; 2.76)	1.46 (0.84; 2.53)
*Organisational variables*		
Procedural justice (ref: no)		
Yes	0.95 (0.71; 1.27)	1.09 (0.82; 1.45)
Part of decision-making processes (ref: no)		
Yes	1.20 (0.95; 1.52)	**1.27 (1.00; 1.60)**
*Relationship with manager*		
Manager who listens (ref: no)		
Yes	1.12 (0.85; 1.47)	1.19 (0.91; 1.56)
Conflict with manager (ref: no)		
Yes	1.00 (0.76; 1.31)	1.02 (0.79; 1.32)
*Coping strategies and motivation*		
Open coping (ref: no)		
Yes	0.84 (0.64; 1.04)	1.13 (0.90; 1.43)
Covert coping (ref: no)		
Yes	1.29 (0.92; 1.82)	0.85 (0.48; 1.51)
Eager to advance (ref: not at all)		
To some extent	1.00 (0.79; 1.26)	0.90 (0.71; 1.13)
Very	**2.12 (1.42; 3.17)**	1.08 (0.68; 1.72)

Values in bold type indicate significant results (p<0.05).

Adjusted for labour market sector and staff category at baseline.

### Human capital variables

Educational level was not associated with promotion among women, but both university education (OR 1.54, 95% CI 1.21; 1.96) and postgraduate education (OR 3.72, 95% CI 1.73; 8.01) significantly predicted an above-average salary increase (see Tables 2 and 3). Among men a postgraduate education predicted promotion and a university education predicted an above-average salary increase (OR 1.59, 95% CI 1.24; 2.04). Working >60 hours/week was significantly related to promotion among women, but weekly working hours were not associated with an above-average salary increase. Among men, working hours were not significantly associated with promotion, but men who worked 51–60 hours/week had higher odds of an above-average salary increase (OR 1.52, 95% CI 1.04; 2.21).

### Organisational variables

As shown in Tables 2 and 3 procedural justice was not related to promotion or an above-average salary increase in either sex. Both women and men who reported to be part of decision-making processes at work had higher odds of being promoted. Taking part in decision-making also predicted an above-average salary increase among men (OR 1.27, 95% CI 1.00; 1.60).

### Relationship with manager

Reporting to have a manager who listened neither predicted promotion (Table 2) nor an above-average salary increase (Table 3) in either sex. Both men and women who reported having conflicts with superiors had higher odds of promotion, but conflicts did not predict an above-average salary increase.

### Coping strategy and motivation

Coping strategies were not associated with career success among women. Men who reported using an open coping strategy when in conflicts with superiors had higher odds of job promotion. Motivation to advance professionally was a significant predictor of promotion in both sexes, and furthermore predicted an above-average salary increase among women (OR 2.12, 95% CI 1.42; 3.17) (see Tables 2 and 3).

### Gender differences

The tests of additive interaction between sex and the other variables indicated two significant differences by sex. Although procedural justice did not predict promotion in either sex, the associations pointed in different directions for men and women and procedural justice was shown to be more important for women’s odds of promotion than men’s (RERI 0.37, 95% CI 0.04; 0.71). Furthermore, men who used an open coping strategy had significantly higher odds of a promotion than women (RERI –0.29, 95% CI –0.58; –0.00). The test of additive interaction furthermore showed that although open coping did not predict an above-average salary increase in either sex, open coping was shown to be more important for men’s odds of a salary increase than for women’s (RERI –0.41, 95% CI –0.71; –0.12). The tests of multiplicative interaction between sex and the other variables in the full regression model yielded no significant associations regarding promotion. However, for above-average salary increase there were significant interaction effects between sex and postgraduate education (p<0.05), very high motivation to advance (p<0.01), and having children living at home (p<0.01) such that women had higher odds of an above-average salary increase, and with using an open coping strategy (p<0.05) such that men had higher odds.

## Discussion

In the present study we found age, education, working hours, taking part in decision-making processes, having conflicts with superiors, and motivation to advance professionally to predict career success in women and men. Several gender differences were observed. Men and women were promoted to the same extent over the study period. However, for women high procedural justice was more important for promotion between 2008 and 2010, and for men using an open coping strategy was more important. Furthermore, men more often than women increased their salaries by more than the average amount between 2008 and 2010, and men had higher odds of an above-average salary increase relative to women when using an open coping strategy. Women had higher odds than men of an above-average salary increase if they had been educated to postgraduate level, had high motivation to advance, and had children living at home. The results are discussed below.

### Socio-demographic variables

Based on previous findings, it was expected that women with children living at home would have a smaller salary increase than men [14,15,21–24]. Contrary to expectations the present results indicated that having children living at home predicted an above-average salary increase among women to a significantly higher extent than among men. However, further analyses showed that the true income raise among mothers was due the fact that their children had grown older and were less sick in 2010 than in 2008.

### Human capital variables

Only men with a postgraduate education had significantly higher odds of promotion, although a trend towards higher odds was seen also among women. The number of hours worked (>60 hours/week) was a significant predictor of promotion among women only, which might be explained by the fact that the relatively few women who worked such long hours became visible to decision-makers [25]. However, in accordance with results reported by Ng et al., no statistical differences between sexes were found in the associations between human capital variables and promotion [13]. Women with a postgraduate education were rewarded in terms of an above-average salary increase to a significantly higher extent than men. It should be noted that a salary increase of >50000 SEK indicates a higher relative increase among women than men. The result may be explained by greater demands on specialised knowledge in fields where women work, such as in health care. Regulations implemented to equalize income differences between sexes may furthermore have favoured women over the time period. However, the result could also indicate that women need better formal merits than men in order to reach higher salary levels, also discussed by Ng et al. [13]. Hours worked predicted an above-average salary increase among men but not women. However, contradictory to results reported by Ng et al. there was no statistically significant gender difference.

### Organisational variables

There was no main effect of high procedural justice on career success for women, which may seem surprising given reports of gender discrimination [26,27]. However, tests of additive interaction revealed that procedural justice may be more important for women to advance, than for men. This indicates that procedural justice may be an important tool if wanting to increase women’s representation on higher organisational levels. Being part of decision-making processes at work predicted job promotion two years later (in both sexes), which may not seem surprising. A promotion may be a natural step following taking part of decision-making processes. Taking part in decision-making was significantly associated with promotion regardless of other factors (e.g. educational level or number of hours worked), which suggests that decision-makers could choose to engage (more) women in decision-making processes if they are interested in promoting more women to higher organisational positions. Men who were part of decision-making processes at work were rewarded in terms of above-average salary increase, which may be explained by beneficial salary trends in male-dominated occupations [12].

### Relationship with manager

Reports of having a manager who listened were not predictive of promotion or an above-average salary increase. In contrast, Ng et al. found supervisor support to be significantly associated with salary [13]. This difference may be due to the fact that slightly different concepts were measured; a manager who listens may not necessarily be supportive. Furthermore, many supervisors may have limited control over their subordinates’ pay, since salaries are often regulated in agreements between employers and unions. The significant association between conflicts with superiors and promotion suggests that individuals who do not shy away from conflicts may be viewed as having leadership skills. The participants’ promotions may also have been preceded by power struggles with a superior that led to successful outcomes for the respondent. The lack of association with an above-average salary increase could be due to more frequent conflicts with superiors in parts of the labour market where salaries are lower [28].

### Coping strategy and motivation

Results of tests for interaction between sex and coping strategy showed that men who used open coping strategies had significantly higher odds of promotion and an above-average salary increase than women (although it was not a significant predictor of an above-average salary increase). This result supports that open coping strategies are associated with better career outcomes for men than women. In previous studies, men have also been shown to use open coping strategies more often than women [18,19]. Agency (e.g., being active and decisive), which theoretically could be linked with open coping strategy, has previously been shown to be associated with career success in both sexes [14,29]. However, the present result suggests that an open communicative style is regarded positively among men, but not women. This result supports the theory of a double bind for women, meaning that women who show competencies associated with successful leadership may be punished for violating gender role expectations [30]. A larger proportion of women than men were not at all eager to advance, which may be explained by lower expectations to prioritize a professional career associated with the female gender role. It could also be due to the fact that in occupations dominated by women possibilities for promotions are very limited, and job promotions may furthermore imply more work but not considerably higher rewards [12]. However, our results also show that women with high career motivation are rewarded with job promotion and higher salaries.

### Strengths and limitations of the study

The present results support previous findings that very highly educated women are better rewarded in terms of salary than men. To our knowledge, it has not previously been shown that a high motivation to advance professionally may predict an above-average salary increase among women more often than among men. Furthermore, the result that women who report a high procedural justice have higher odds of a promotion than men is important. It indicates that improving procedural justice in organisations has the potential to increase the female representation in more senior roles. The finding of a gender difference in positive career outcomes associated with using an open coping strategy is also interesting. It supports the existence of strong gender role expectations in behaviours also in a country, Sweden, which has relatively high gender equality. However, there are several limitations to take into account when interpreting our results. The participants were followed for only two years, and the long-term effects of career interruptions, known to strongly impact career success, were not evaluated [14,21,24,31]. There are also several other possible predictors of promotion not included in this study, e.g., mentoring and further training, because no such data were available in SLOSH. Another limitation is that we did not have data regarding which organisational levels the participants were working at. Furthermore, many of the variables used represent the perception of the individuals, which may be influenced by the dependent variable of promotion. However, because the predictors were measured before promotion, there is limited risk that the results are influenced by an erroneously positive or negative perception of, for example, procedural justice depending on promotion. Furthermore, data on salaries were taken from national registers and could thereby not be influenced by other factors measured in the study. As mentioned above, the study is derived from a nationally representative sample, but due to attrition bias there are some limitations in the generalizability of our results. There was, for example, a lower representation of younger age groups in this study and therefore we cannot fully generalise our results to the youngest Swedish workers. In spite of these limitations, the results add considerably to the literature by assessing gender differences in predictors of career success in prospective data derived from a nationally representative sample in a country that has relatively high gender equality.

### Conclusion

The present prospective study supports previous results showing gender differences in human capital predictors of salary. Our results indicate that women need to attain higher educational credentials and have a stronger career ambition than men in order to obtain an above-average salary increase. Women furthermore appear to be more dependent on organisational characteristics (justice) in order to advance than men. Men were more successful in their careers than women when they exhibited typical male attributes (such as being active and decisive). Our results support the existence of gendered structures in Swedish working life, where women rely more on formal knowledge, ambition, and justice within the organisation to be successful and men rely more on behavioural components related to their gender role.
